# Special Issue: Materials for Nuclear Waste Immobilization

**DOI:** 10.3390/ma12213611

**Published:** 2019-11-03

**Authors:** Neil C. Hyatt, Michael I. Ojovan

**Affiliations:** 1 Immobilisation Science Laboratory, Department of Materials Science and Engineering, University of Sheffield, Mappin Street, Sheffield S1 3JD, UK; n.c.hyatt@sheffield.ac.uk; 2 Department of Radiochemistry, Lomonosov Moscow State University, Moscow 119991, Russia; 3 Imperial College London, South Kensington Campus, Exhibition Road, London SW7 2AZ, UK

**Keywords:** nuclear waste, spent nuclear fuel, immobilisation, conditioning, wasteforms, vitrification, glass, ceramics, glass composite materials, durability

## Abstract

Nuclear energy is clean, reliable, and competitive with many useful applications, among which power generation is the most important as it can gradually replace fossil fuels and avoid massive pollution of environment. A by-product resulting from utilization of nuclear energy in both power generation and other applications, such as in medicine, industry, agriculture, and research, is nuclear waste. Safe and effective management of nuclear waste is crucial to ensure sustainable utilization of nuclear energy. Nuclear waste must be processed to make it safe for storage, transportation, and final disposal, which includes its conditioning, so it is immobilized and packaged before storage and disposal. Immobilization of waste radionuclides in durable wasteform materials provides the most important barrier to contribute to the overall performance of any storage and/or disposal system. Materials for nuclear waste immobilization are thus at the core of multibarrier systems of isolation of radioactive waste from environment aimed to ensure long term safety of storage and disposal. This Special Issue analyzes the materials currently used as well as novel materials for nuclear waste immobilization, including technological approaches utilized in nuclear waste conditioning pursuing to ensure efficiency and long-term safety of storage and disposal systems. It focuses on advanced cementitious materials, geopolymers, glasses, glass composite materials, and ceramics developed and used in nuclear waste immobilization, with the performance of such materials of utmost importance. The book outlines recent advances in nuclear wasteform materials including glasses, ceramics, cements, and spent nuclear fuel. It focuses on durability aspects and contains data on performance of nuclear wasteforms as well as expected behavior in a disposal environment.

Materials are at the core of multibarrier systems of isolation of radioactive (nuclear) waste from the environment. Relevant materials are used to ensure long-term safety of handling, storage, transportation, and disposal of nuclear waste. Nuclear waste immobilization is the conversion of waste into a wasteform by solidification, embedding, or encapsulation that reduces the potential for migration or dispersion of radionuclides during operational and disposal stages of waste lifecycle. Immobilization of waste is achieved by its chemical incorporation into the structure of a suitable matrix (typically cement, glass, or ceramic) so it is captured and unable to escape. Chemical immobilization is typically applied to high level waste (HLW). Encapsulation of waste is achieved by physically surrounding it in materials (typically bitumen or cement) so it is isolated, and radionuclides are retained. Physical encapsulation is often applied to intermediate level waste (ILW) but can also be used for HLW, especially where chemical incorporation of radionuclides in the surrounding matrix is also possible.

Within the repository, the wasteform is one part of a multiple engineered barrier system. During storage and transportation, the wasteform is the primary barrier preventing the release of radionuclides into the environment, while during post closure disposal, the wasteform will reduce the release of radionuclides from breached and compromised containers that could result due to corrosion, earthquake, human intrusion, igneous intrusion (volcano), or other disruptive phenomena.

Choosing a suitable wasteform (matrix) to use for nuclear waste immobilization is not easy and its durability is not the sole acceptance criterion. Priority is given to reliable, simple, rugged technologies and equipment, which may have advantages over complex or sensitive equipment and processes. A variety of matrix materials and techniques are available for immobilisation [1,2,3,4,5,6,7,8,9,10,11,12,13,14,15,16]. The choice of the immobilization technology depends on the physical and chemical nature of the waste and the acceptance criteria for the storage and disposal facility to which the waste will be consigned.

Factors that are considered primarily when selecting a wasteform material are as follows [3,4]:

- Waste loading—able to accommodate a significant amount of waste (typically 25–45 weight %) to minimize volume;
- Ease of production—accomplished under reasonable conditions;
- Durability—low rate of dissolution to minimize the release of radioactive and chemical constituents;
- Radiation stability—high tolerance to radiation effects from the decay of radioactive constituents;
- Chemical flexibility—able to accommodate a mixture of radioactive and chemical constituents with minimum formation of secondary phases;
- Availability of natural analogues—availability of natural mineral or glass analogues may provide important clues about the long-term performance;
- Compatibility with the intended disposal environment—compatible with the near-field environment of the disposal facility.

A host of regulatory, process, and product requirements has led to the investigation and adoption of a variety of matrices and technologies for waste immobilization. The resistance of the wasteform to aqueous corrosion and release of radionuclides in the disposal environment—chemical durability—is a critical parameter. Figure 1 shows schematically the water durability of main nuclear wasteforms used.

The main immobilization technologies that are available commercially and have been demonstrated to be viable are cementation [1,2,3,4,5] and vitrification [1,3,4,8,9,10,11,12,13,14,15,16], whereas bitumen and polymeric materaisl are used to a smaller extent (see data in [1]) and ceramification is a perspective technology [1,3,6,7,8,12,14,15,16]. Table 1 shows generic features and limitations of main wasteforms currently used on industrial scale.

The general requirements against one another need optimization for any technological approach considered. For example, ceramics are credited with having higher chemical durability than glasses, however, radionuclides will be released at similar or even higher rates compared with glassy wasteforms (see, e.g., Figure 1) if they are incorporated in the lower durability crystalline phases and intergranular glassy phases.

This book contains 10 dedicated papers prepared by lead researchers covering different aspects of nuclear wasteforms and their expected behavior. They purposely analyze the materials currently used as well as novel materials for nuclear waste immobilization including technological approaches utilized in nuclear waste conditioning pursuing to ensure efficiency and long-term safety of storage and disposal systems, including cementitious materials, glasses, and ceramics. The book outlines recent advances in nuclear wasteform materials including cements, glasses, ceramics, cements, and spent nuclear fuel with focus on durability aspects and presenting data on performance of nuclear wasteforms, as well as expected behavior in a disposal environment.

## Figures and Tables

**Figure 1:**
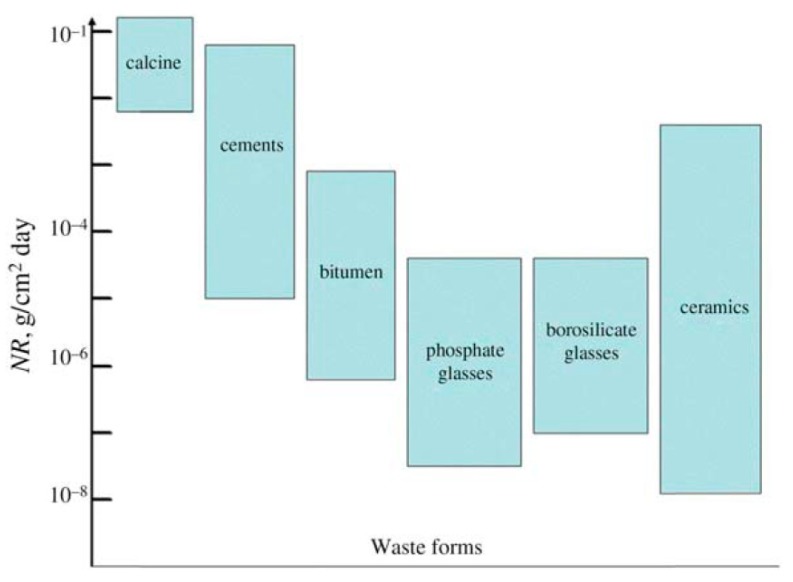
Normalized leaching rates of various wasteforms (after Reference [1]).

**Table 1:** Features and limitations of main wasteforms currently used.

Wasteform	Features	Limitations	Secondary Waste
Glasses	Proven method to condition liquid high-level waste (HLW) as well as intermediate-level waste (ILW) and low-level waste (LLW). High flexibility in terms of the glass formulation range. High reliability of the immobilization process. High glass throughput. High durability of the final wasteform. Small volume of the resulting wasteform.	High initial investment and operational costs. Complex technology requiring high qualified personnel. Need to control off-gases. High specific energy consumption.	Off-gases. Filters. Scrub solutions. Used melters.
Ceramics	Possible to incorporate higher levels of actinides than borosilicate glass. Wasteform can be more durable than glass. Expected to be suitable for long term isolation since it simulates natural rocks.	Limited experience. Most efforts have been research-based. The ceramic shall be tailored to nuclear waste composition.	Filters. Off-gases. Scrub solutions.
Glass-composite materials	Combine features of both crystalline and glassy materials. Higher waste loading. Higher compatibility. Higher stability compared glasses.	Limited experience.	Off-gases. Filters. Scrub solutions. Used melters
Cements	Widely used method for variety of LLW and ILW. High flexibility. Low cost. Simplicity of process. Low temperature precludes volatile emissions. High radiation stability, impact, and fire resistance of wasteforms.	Increase of volume (low waste loading). Low retention of some fission and activation products. Poor compatibility with organic materials and high-salt content.	None.
Bitumen	Mostly used for LILW, chemical precipitates, low heat, and low alpha wastes. High flexibility. High compatibility with organic materials. High waste loading. Lower leaching rate compared with cements.	Sensitivity to some components. Low fire resistance.	Filters.
Metals	Extensively proven technology for conditioning of metallic waste. The product is typically homogeneous and stable.	Pre-sorting is usually required due to dedicated melt furnaces and differences in melt temperatures of different metals.	Off-gases. Slag.
